# Bone marrow sympathetic neuropathy is a hallmark of hematopoietic malignancies and it involves severe ultrastructural damage

**DOI:** 10.1186/s40164-025-00614-x

**Published:** 2025-03-05

**Authors:** Aurora Bernal, Vincent Cuminetti, Marc Serulla, Adrian Florit, Joanna Konieczny, Golnaz Golnarnik, Yimeng Chen, Marc Ferré, Samuel Geiseler, Anders Vik, Randi Olsen, Lorena Arranz

**Affiliations:** 1 Stem Cells, Ageing and Cancer Research Group, Department of Medical Biology, Faculty of Health Sciences, UiT – The Arctic University of Norway, MH2 Building Level 10, 9019 Tromsø, Norway; 2 Stem Cells, Ageing and Cancer Research Group, Centre of Embryology and Healthy Development, Institute of Clinical Medicine, Faculty of Medicine, University of Oslo, 0373 Oslo, Norway; 3 Department of Hematology, University Hospital of North Norway, 9019 Tromsø, Norway; 4 Department of Clinical Medicine, Faculty of Health Sciences, UiT – The Arctic University of Norway, 9019 Tromsø, Norway; 5 Advanced Microscopy Core Facility, Department of Medical Biology, Faculty of Health Sciences, UiT – The Arctic University of Norway, MH2 Building Level 9, 9019 Tromsø, Norway; 6 Associate Investigator, Norwegian Center for Molecular Medicine (NCMM), University of Oslo, 0349 Oslo, Norway

**Keywords:** Stem cell niche, Hematological cancers, Peripheral nervous system, Sympathetic fibres, Transmission electron microscopy

## Abstract

The hematopoietic stem cell (HSC) niche in the bone marrow (BM) supports HSC function, fate and numbers [1]. Sympathetic fibres innervate the BM and are components of the hematopoietic stem and progenitor cell (HSPC) niche [2]. Neuropathy of the HSPC niche is present and essential for disease development in experimental models of *JAK2*^*V617F*+^ myeloproliferative neoplasms (MPN) and *MLL-AF9*^+^ acute myeloid leukemia (AML), and it is present in the BM of human MPN and AML patients [3–6]. Neuropathy contributes to mutant HSC expansion and represents an effective therapeutic target to block disease progression in *JAK2*^*V617F*+^ MPN mice [3]. The sympathomimetic agonist mirabegron restored nestin^+^ cells and reduced reticulin fibrosis in MPN patients [7]. Here, we show that neuropathy of the HSPC niche emerges in two additional experimental models of hematological disease including pre-leukemic myelopoiesis driven by *NRAS*^*G12D*^ and lymphoma/lymphoblastic leukemia driven by *p53* deletion. Neuropathy involves severe ultrastructural damage in *NRAS*^*G12D*+^ mice and AML patients as shown by electron microscopy. When further reinforced chemically, neuropathy has a profound impact on the experimental *NRAS*^*G12D*^ mouse model, promoting myeloid bias, reducing HSPC numbers and inducing changes in the stem cell microenvironment that include reduced numbers of mesenchymal stromal cells (MSC) and increased presence of morphologically abnormal blood vessels in BM. Together, BM neuropathy is a prevalent factor in hematopoietic malignancies that involves important degradation of sympathetic fibres and contributes to disease in a different manner depending on the driver mutation. This should be taken in consideration in the clinic, given that chemotherapy induces neuropathy of the HSC niche [8] and it is the most frequent first line treatment for AML, acute lymphoblastic leukemia and MPN patients.

**Supplementary Information:**

The online version contains supplementary material available at 10.1186/s40164-025-00614-x.

## Main text

**Fig. 1:**
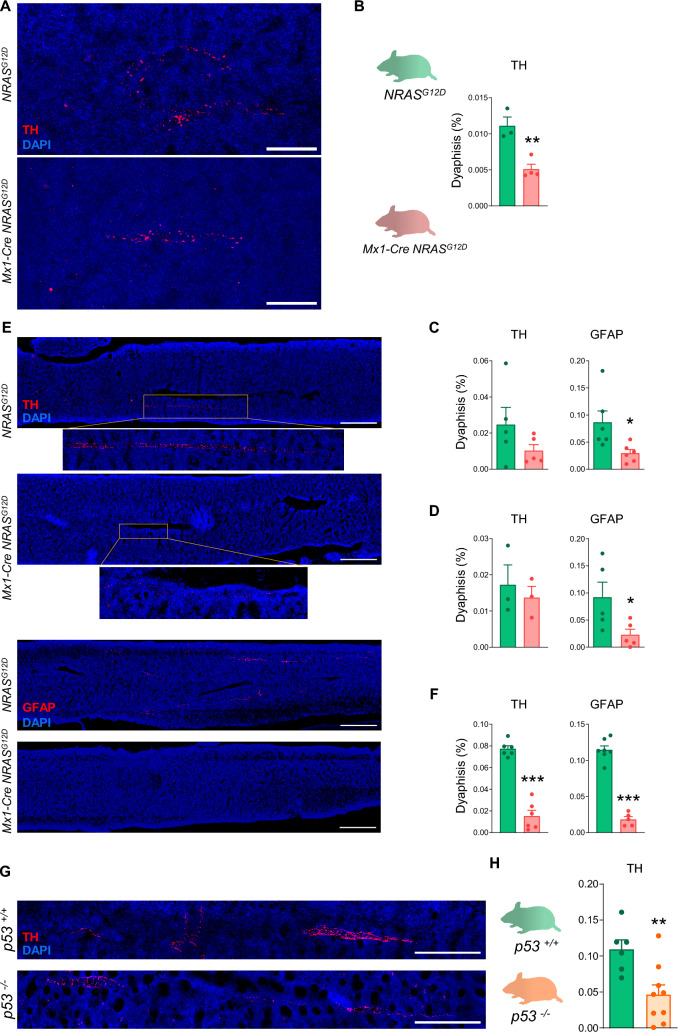

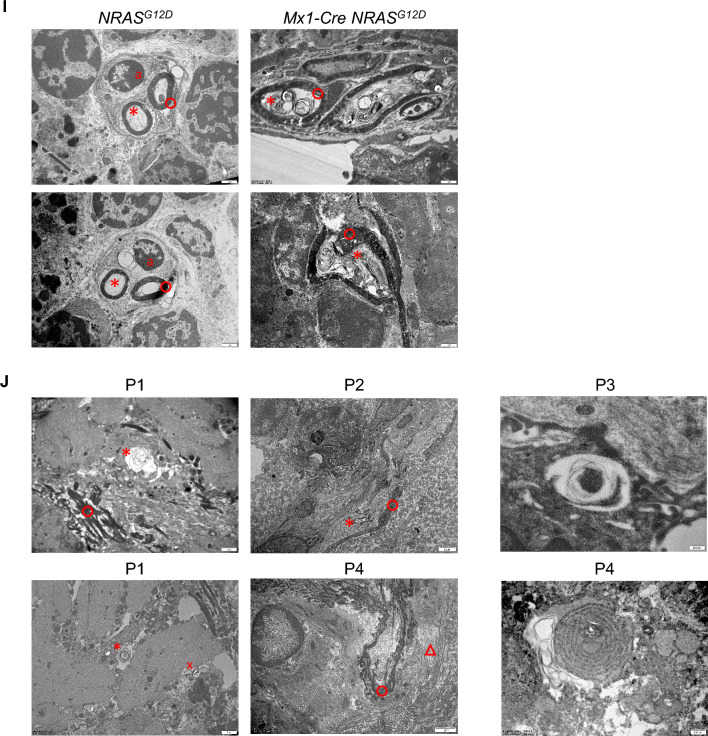
Bone marrow neuropathy is a hallmark of disease in mouse models of both myeloid and lymphoid malignancies, and it involves severe ultrastructural damage to myelin and axons in pre-leukemic NRAS-G12D^+^ primary mutant mice and acute myeloid leukemia (AML) patients. C57BL/6J female mice transplanted with bone marrow (BM) nucleated cells obtained from polyinosine-polycytosine (pI-pC)-induced control (*NRAS*^*G12D*^) or NRAS-G12D^+^ (*Mx1-Cre NRAS*^*G12D*^) mice, and analyzed at 40 weeks of age, 32 weeks after the transplant (n = 3–4 per group). (**A**) Representative immunostaining of tyrosine hydroxylase (TH, red) to visualize sympathetic fibres in BM; nuclei were counterstained with DAPI (blue); scale bar, 100 µm. (**B**) Quantification of TH^+^ sympathetic fibres in BM diaphysis (%). (**C-F**) Time-course analysis of TH^+^ sympathetic fibres and of glial fibrillary acidic protein (GFAP)^+^ ensheathing Schwann cells in BM diaphysis from control (*NRAS*^*G12D*^) or NRAS-G12D^+^ (*Mx1-Cre NRAS*^*G12D*^) primary mutant female mice, induced with pI-pC at 7–27 weeks of age. (**C**) Quantification of TH^+^ sympathetic fibres and of GFAP^+^ ensheathing Schwann cells in BM diaphysis (%), 6 weeks after pI-pC induction (n = 5–6 per group). (**D**) Quantification of TH^+^ sympathetic fibres and of GFAP^+^ ensheathing Schwann cells in BM diaphysis (%), 24 weeks after pI-pC induction (n = 3–5 per group). (**E**) Representative immunostaining of TH (red, upper panel) to visualize sympathetic fibres and of GFAP (red, lower panel) to visualize ensheathing Schwann cells in BM; nuclei were counterstained with DAPI (blue); scale bar, 500 µm. Magnification is shown for better appreciation of TH^+^ sympathetic fibres, and (**F**) Quantification in BM diaphysis (%), 38 weeks after pI-pC induction (n = 5–7 per group). (**G-H**) Wild-type (*p53*^+/+^) or *p53*^−/−^ female and male mice, analyzed at 12 weeks of age (n = 6–9 per group). (**G**) Representative immunostaining of TH (red, upper panel) to visualize sympathetic fibres in BM; nuclei were counterstained with DAPI (blue); scale bar, 250 µm. (**H**) Quantification in BM diaphysis (%). *p < 0.05, **p < 0.01, ***p < 0.001 unpaired two-tailed t test. (**I**) Representative transmission electron microscopy (TEM) images of GFAP^+^ BM sections from control (*NRAS*^*G12D*^) or NRAS-G12D^+^ (*Mx1-Cre NRAS*^*G12D*^) male mice, analyzed at 29 weeks of age, 20 weeks after pI-pC induction (n = 2–3 per group). (**J**) Representative TEM images of GFAP^+^ BM sections from AML patients (n = 4, P1-P4). a, Schwann cell nucleus; o, myelin sheath; *, axon; △, collagen fibres; x, tubule-vesicular elements. Scale bar indicated in the images.

To the editor,

We first made use of the *Mx1-Cre NRAS*^*G12D*^ mouse model of aberrant pre-leukemic myelopoiesis [9], from now on referred to as NRAS-G12D^+^. Cre recombinase was activated by administration of 300 µg of polyinosine-polycytosine (pI-pC) via intraperitoneal route at 8 weeks of age. Mice were sacrificed at 20 weeks of age, 12 weeks after induction, and their BM nucleated cells were extracted and transplanted into recipient mice. Age-matched, female wild-type C57BL/6J mice were used as recipients in the BM transplantation assay. Recipient mice were preconditioned at 8 weeks of age with the chemotherapeutic drug busulfan. One dose of 25 mg/kg was administered via intraperitoneal route. 24 h after, 2 × 10^6^ BM nucleated cells previously obtained from pI-pC-induced NRAS-G12D^+^ (*Mx1-Cre NRAS*^*G12D*^) or control (*NRAS*^*G12D*^) mice were injected intravenously to recipients. At 32 weeks after the transplant, animals were sacrificed and BM sections were used for immunofluorescence of tyrosine hydroxylase (TH)^+^ sympathetic nerve fibres, which were markedly reduced in the experimental *Mx1-Cre NRAS*^*G12D*^ model (Fig. 1A, B).

Chemotherapy induces neuropathy of the HSC niche in the mice [8]. To rule out any potential confounding effect of busulfan, we then used *Mx1-Cre NRAS*^*G12D*^ primary mutant mice versus *NRAS*^*G12D*^ control mice for the study of TH^+^ sympathetic fibres and ensheathing glial fibrillary acidic protein (GFAP)^+^ Schwann cells in the BM. One of the main drawbacks of *Mx1-Cre NRAS*^*G12D*^ mouse model is that it is inducible with an inflammatory signal that may have effects on HSC and on components of the microenvironment. However, BM isolated from mice treated three times with similar doses of pI-pC (followed by 10 days recovery) was serially transplanted into irradiated recipients and no significant difference in the repopulation activity of pI-pC treated cells was observed [10]. The authors concluded that this transient activation of IFN-α signaling does not affect the number of functional HSC, as opposed to chronic activation of this pathway. To reduce any potential confounding effect derived from pI-pC-induced inflammation and still analyze an early disease stage with most primitive HSC showing highest expansion, mice were monitored starting 6 weeks post pI-pC induction, and controls used were *NRAS*^*G12D*^ with no Cre treated with pI-pC as previously described. Adult female mice (7–27 weeks) were induced with one or two injections of pI-pC as described above, and analyzed 6, 24 and 38 weeks after. Time-course analysis showed that BM damage to Schwann cells happens early in the course of the disease and is maintained over time. Further, BM damage to Schwann cells precedes neural damage to sympathetic fibres (Fig. 1C–F).

As an independent model of hematopoietic malignancy, we used *p53*^*−/−*^ mice that develop lymphoma/lymphoblastic leukemia (87%) and not AML [11]. Male and female mice were sacrificed at 12 weeks of age and BM sections were used for immunofluorescence of TH^+^ sympathetic nerve fibres. Sympathetic fibres were markedly reduced in the BM of *p53*^*−/−*^ mice versus wild-type mice (Fig. 1G, H). These results indicate that neuropathy of the BM is not restricted to myeloid leukemias but it is a common event in hematopoietic malignancies.

We aimed at visualizing and providing a better understanding of the ultrastructure of BM sympathetic fibres. For this purpose, male *Mx1-Cre NRAS*^*G12D*^ or *NRAS*^*G12D*^ control mice were induced at 9 weeks of age with 300 µg of pI-pC and sacrificed 20 weeks after. Immunofluorescence for GFAP staining was used to locate the fibres in the BM to proceed with transmission electron microscopy (TEM). Samples were then processed and embedded for TEM, which was followed by ultrathin sectioning. Post-analysis of BM neural fibres in control mice using TEM showed regular structures of axon and myelin sheath (Fig. 1I). Instead, the structure of axon and myelin sheath in NRAS-G12D^+^ pre-leukemic mice were disorganized, and severe axonal demyelination and degeneration were uncovered. Demyelination was shown by thickened and less dense myelin sheath of sympathetic fibres in the BM of NRAS-G12D^+^ pre-leukemic versus control mice, and the presence of products of organelles, dense bodies, vacuoles and loops of myelin debris in the axoplasm accounted for axonal degeneration (Fig. 1I). The severity of the disruption in the integrity of the BM sympathetic fibres was reminiscent of neurodegenerative diseases like Duchenne muscular dystrophy [12].

AML patients show BM sympathetic neuropathy [6] and we used the same technique specified above in BM biopsies from AML patients (n = 4) to study the ultrastructure of BM sympathetic fibres. Written informed consent was obtained in accordance with the Norwegian legislation and the Declaration of Helsinki. The study was approved by the *Regional komité for medisinsk og helsefaglig forskningsetikk REK Nord-Norge* (REK nord 2015/1082). The diagnosis of AML was established according to the revised criteria of the World Health Organization. Cytogenetic risk group was established according to Dohner and colleagues [13]. Myeloblasts were calculated by morphological evaluation of BM smears, complemented by regular flow cytometry. Individual characteristics of AML patients are shown in Supplementary Table S1. The structure of axon and myelin sheath in the BM of all AML patients studied were severely disorganized, and axonal demyelination and degeneration was evident (Fig. 1J). The BM of patient P1 showed axons with remarkable dystrophic collection of compacted tubule-vesicular elements. BM collagen fibrosis accompanied by severe uncompacted myelin was detected in patient P4 (Fig. 1J). Thus, BM neuropathy with presence of severe degeneration of the ultrastructure of BM sympathetic fibres is consistent in AML patients, despite AML patient heterogeneity. Future studies increasing the sample size aimed at uncovering potential differences in rate, degree or dynamics of the BM neuropathy depending on the driver mutation, and other factors such as the age of the patient, will be highly relevant.

**Fig. 2:**
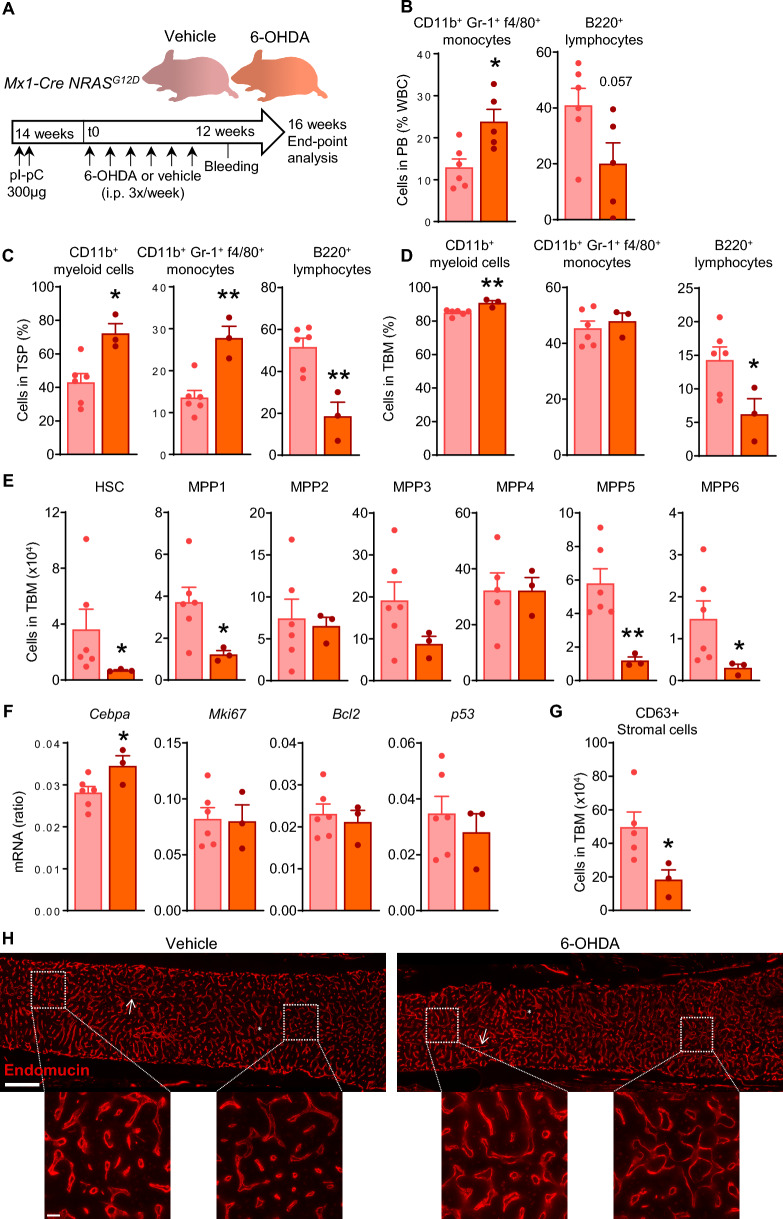
Enforced neuropathy worsens pre-leukemic phenotype in NRAS-G12D^+^ primary mutant mice. (**A**) In vivo intraperitoneal (i.p.) administration with 6-hydroxydopamine (6-OHDA) to induce chemical sympathectomy or vehicle in NRAS-G12D^+^ (*Mx1-Cre NRAS*^*G12D*^) female mice aged 25–49 weeks for a total of 16 weeks, starting 14 weeks after polyinosine-polycytosine (pI-pC) induction. (**B**) Fraction of CD11b^+^Gr-1^+^f4/80^+^ monocytes and B220^+^ B lymphocytes in total white blood cells (WBC) analyzed by fluorescence-activated cell sorting (FACS), after 12 weeks of treatment (n = 5–6 per group). (**C**) Fraction of CD11b^+^ myeloid cells, monocytes and B lymphocytes in total spleen cells (TSP), analyzed by FACS, after 16 weeks of treatment (n = 3–6). (**D**) Fraction of CD11b^+^ myeloid cells, monocytes and B lymphocytes in bone marrow nucleated cells (BMNC) analyzed by FACS, after 16 weeks of treatment (n = 3–6). (**E**) Total BM (TBM) number of Lin^−^c-Kit^+^Sca-1^+^ (LSK) cell subsets: LSK CD34^−^Flt3^−^CD48^−^CD150^+^, hematopoietic stem cells (HSC); LSK CD34^+^Flt3^−^CD48^−^CD150^+^, multipotent progenitors 1 (MPP1); LSK CD34^+^Flt3^−^CD48^+^CD150^+^ (MPP2); LSK CD34^+^Flt3^−^CD48^+^CD150^−^ (MPP3); LSK CD34^+^Flt3^+^CD48^+^CD150^−^ (MPP4); LSK CD34^+^Flt3^−^CD48^−^CD150^−^ (MPP5); LSK CD34^−^Flt3^−^CD48^−^CD150^−^ (MPP6) analyzed by FACS, after 16 weeks of treatment (n = 3–6). (**F**) Gene expression analysis of *Cebpa*, *Mki67*, *Bcl2* and *p53* relative to *Gapdh* by digital droplet PCR after reverse transcription of total mRNA extracted from FACS-sorted LSK, after 16 weeks of treatment (n = 3–6). (**G**) TBM number of CD45^−^CD31^−^Ter-119^−^CD63^+^ mesenchymal stromal cells analyzed by FACS, after 16 weeks of treatment (n = 3–5). (**H**) Representative immunostaining of endomucin (red) to visualize sinusoids and arterioles in BM, 16 weeks after treatment; scale bar, 500 µm. Magnification is shown for better appreciation of blood vessels; scale bar, 50 µm (n = 2–4). *, sinusoids; arrows, arterioles. *p < 0.05, **p < 0.01 unpaired two-tailed t test

In an MLL-AF9^+^ AML model, generated after transduction of Lin^−^c-Kit^+^Sca-1^+^ (LSK) cells with the MLL-AF9 oncogene and serial transplantation of these cells, Hanoun et al. showed that neuropathy is a hallmark of disease and it promotes leukemic BM infiltration [4]. To check if BM neuropathy contributes to disease in the pre-leukemic *Mx1-Cre NRAS*^*G12D*^ model, we reinforced it using a similar strategy of chemical sympathectomy in female NRAS-G12D^+^ (*Mx1-Cre NRAS*^*G12D*^) mice aged 25–49 weeks, by intraperitoneal injection with 100 mg/kg of 6-hydroxydopamine (6-OHDA) prepared in oxygen-free PBS supplemented with sodium metabisulfite at 1 mg/ml. The treatment was administered on alternate days for 16 weeks, starting 14 weeks after pI-pC induction with two injections in consecutive days (Fig. 2A). NRAS-G12D^+^ mice show pre-leukemic myelopoiesis by means of increased circulating monocytes and decreased B lymphocytes compared to control mice [14], and a 12-week treatment with 6-OHDA aggravated NRAS-G12D^+^ pre-leukemic phenotype in peripheral blood (PB) (Fig. 2B and Supplementary Fig. 1A). Extramedullary hematopoiesis in spleen was significantly increased after 16 weeks of 6-OHDA treatment in NRAS-G12D^+^ mice, with higher myeloid cells, particularly monocytes, at the expense of B lymphocytes (Fig. 2C). In BM, 6-OHDA administration increased myeloid cell frequency and further decreased the low B cell frequency (Fig. 2D). Analysis of the hematopoietic stem and progenitor cell (HSPC) subsets corresponding to hematopoietic stem cells (HSC) and multipotent progenitors MPP1-MPP6 [15] revealed a selective decrease of the low numbers of HSC, accompanied by reductions in MPP1, MPP5 and MPP6, in mice treated with 6-OHDA compared to vehicle (Fig. 2E and Supplementary Fig. 1B). To understand these functional changes, we FACS-sorted BM LSK cells from vehicle or 6-OHDA treated mice and performed gene expression analysis by digital droplet PCR (ddPCR) relative to *Gapdh* (Fig. 2F). We studied *Cebpa* gene expression as a marker of myeloid differentiation, and found it significantly increased in LSK cells from the BM of NRAS-G12D^+^ mice treated with 6-OHDA versus vehicle. There were no differences in the expression of *Mki67* as a marker of proliferation, or in the expression of *Bcl2* and *p53* as representative regulators of apoptosis.

NRAS-G12D^+^ mice show damage to the BM stroma, including decreased numbers of BM CD63^+^ stromal cells compared to control mice, which contribute to abnormal myelopoiesis [14]. To investigate potential sympathectomy-induced damage to the stromal compartment, we digested the bones with collagenase and analyzed the numbers of CD63^+^ MSC within the CD45^−^CD31^−^Ter-119^−^ non-hematopoietic fraction of the BM by FACS, as previously described [3, 14]. 6-OHDA treatment significantly reduced the number of BM CD63^+^ MSC in NRAS-G12D^+^ compared to vehicle-treated mice. Together, this suggests that BM neuropathy in NRAS-G12D^+^ pre-leukemic mice may contribute to NRAS-G12D-driven chronic pathogenesis and supports NRAS-G12D as a cooperative mutation, by promoting myelopoiesis and damage to the stromal microenvironment by means of CD63^+^ MSC in the BM.

It is important to consider that pI-pC induced NRAS-G12D mutation could potentially affect non-hematopoietic tissues in the *Mx1-Cre NRAS*^*G12D*^ mouse model. However, pathologic sections of the BM and spleen of moribund animals at the age of 48–60 weeks, comparable to those used in the present work, revealed underlying myeloproliferation regardless of the assigned cause of death [16, 17]. Therefore, it is unlikely that the observed changes after 6-OHDA treatment could be related to NRAS-G12D expression in non-hematopoietic tissues, but this possibility may not be ruled out from our work and requires further investigation.

Here, we show that BM neuropathy may be an early event in the development of *NRAS*^*G12D*+^ MPN, similarly to *JAK2*^*V617F*^^+^ MPN [3]. As mentioned above, previous work showed that acute chemical sympathectomy increases infiltration of LSK cells transduced with MLL-AF9 oncogene after transplantation in an aggressive course of experimental AML and leukemic MLL-AF9^+^ cells induce sympathetic neuropathy in infiltrated sites [4]. Unlike MPN [3], AML development led to an expansion of MSC, which were primed for osteoblastic differentiation [4]. Chronic chemical sympathectomy in the pre-leukemic NRAS-G12D^+^ experimental model further increased myeloid expansion in BM and periphery, with loss of HSC and early multipotent progenitors MPP1, MPP5 and MPP6 in BM, and reduced numbers of BM CD63^+^ MSC. Thus, neural disruption of the HSC niche contributes to progression of NRAS-G12D^+^ disease and exhaustion of the HSC pool. Although future functional studies should be warranted, our work at the transcription level of selected genes suggests that LSK exhaustion may be driven by increased differentiation to the myeloid lineage with unchanged proliferation or apoptosis. Neuropathy may be a relevant factor contributing to the chronic nature of the hematopoietic abnormalities displayed by NRAS-G12D^+^ mice, which require a second mutation to transform into acute leukemia [18]. Importantly, BM neuropathy contributes to disease in a different manner depending on the driver mutation.

We previously linked IL-1β–induced damage to the neural and subsequently to the MSC components of the HSC niche to MPN pathogenesis in mice [3]. Neuropathy and aberrant expression of IL-1β were concomitant in the BM of patients with JAK2-V617F^+^ essential thrombocythemia [5]. Our previous data demonstrated enhanced IL-1β signaling as a main underlying mechanism contributing to CD63^+^ MSC apoptosis and disease progression in NRAS-G12D^+^ pre-leukemic mice [14]. Here, we show that neuropathy is a prevalent factor that contributes to reduce CD63^+^ MSC numbers and promotes disease progression in the same pre-leukemic model. The sequence of events involving neuropathy, stromal cell damage and disease progression as well as the potential link to IL-1β should be subject of future studies. Interestingly, IL-1β has been associated to non-myeloid, lymphoma/lymphoblastic leukemia [19], and we provide evidence that neuropathy is present in lymphoma/lymphoblastic leukemia driven by p53 deletion. Future studies should further explore the causal link and potential synergistic effect of IL-1β–induced inflammation and neuropathy of the HSC niche in the pathogenesis of lymphoid malignancies.

Cross-talk between leukemic and endothelial cells in the BM stem cell microenvironment promotes angiogenesis, which in turn predicts poor prognosis in AML [20]. To study potential sympathectomy-induced changes in BM vessels, BM sections were used for immunofluorescence of endomucin^+^ sinusoids and arterioles [21]. Endomucin^+^ sinusoids displayed abnormal morphologies and their number per mm^2^ were expanded by 20% (n = 2–3) in the BM of 6-OHDA-treated NRAS-G12D^+^ mice compared to vehicle-treated mice (Fig. 2H). Thus, BM neuropathy contributes to complex changes in the stem cell microenvironment with potential to impact the course of the hematological malignancy that will require future investigation.

Together, BM neuropathy may be a common event not restricted to myeloid malignancies that involves severe damage of sympathetic fibres at the ultrastructural level and contributes to disease in various ways, which are dependent on the specific driver mutation. BM neuropathy in hematopoietic malignancies may contribute to further complications as it has previously been linked to retinopathy in diabetes [22]. Given that chemotherapy induces neuropathy of the HSC niche [8] and is the most frequent first line treatment for patients with acute leukemias and chronic neoplasms, its use should be evaluated with caution on an individual basis. Further, although in healthy conditions, the peripheral nervous system retains capacity to regenerate after injury, it will be important to investigate the degree of regeneration after curative strategies against hematopoietic malignancies or potential long-lasting effects that could impact HSC and progenitor function.

## Supplementary Information

**Additional file 1.** Supplementary Fig. 1 (related to Fig. 3). Representative dot plots used for fluorescence-activated cell sorting (FACS) analyses. (A) Representative FACS analyses and fractions of cells in white blood cells, of CD11b^+^ myeloid cells, CD11b^+^Gr-1^+^f4/80^+^ monocytes and B220^+^ B lymphocytes of one control mouse. (B) Representative FACS analyses and fractions of cells in total bone marrow (TBM) of Lin^−^ and Lin^−^c-Kit^+^Sca-1^+^ (LSK) cell subsets: LSK CD34^−^Flt3^−^CD48^−^CD150^+^, hematopoietic stem cells (HSC); LSK CD34^+^Flt3^−^CD48^−^CD150^+^, multipotent progenitors 1 (MPP1); LSK CD34^+^Flt3^−^CD48^+^CD150^+^ (MPP2); LSK CD34^+^Flt3^−^CD48^+^CD150^−^ (MPP3); LSK CD34^+^Flt3^+^CD48^+^CD150^−^ (MPP4); LSK CD34^+^Flt3^−^CD48^−^CD150^−^ (MPP5); LSK CD34^−^Flt3^−^CD48^−^CD150^−^ (MPP6) of one control mouse. (C) Representative FACS analysis and fractions of cells in TBM of CD45^−^CD31^−^Ter-119^−^CD63^+^ mesenchymal stromal cells of one control mouse.

**Additional file 2.** Table S1. Individual characteristics of acute myeloid leukemia patients.

## Data Availability

Further information and requests for resources and reagents should be directed to and will be fulfilled by the corresponding author, L. Arranz (lorena.arranz@medisin.uio.no).
