# Supplementary material for: Bone marrow sympathetic neuropathy is a hallmark of hematopoietic malignancies and it involves severe ultrastructural damage
Source: Exp Hematol Oncol. 2025 Mar 5;14:31. doi: 10.1186/s40164-025-00614-x (PMC11884145; doi:10.1186/s40164-025-00614-x)
Supplement: Supplementary file 1 — Additional file 1. Supplementary Fig. 1 (related to Fig. 3). Representative dot plots used for fluorescence-activated cell sorting (FACS) analyses. (A) Representative FACS analyses and fractions of cells in white blood cells, of CD11b+ myeloid cells, CD11b+Gr-1+f4/80+ monocytes and B220+ B lymphocytes of one control mouse. (B) Representative FACS analyses and fractions of cells in total bone marrow (TBM) of Lin− and Lin−c-Kit+Sca-1+ (LSK) cell subsets: LSK CD34−Flt3−CD48−CD150+, hematopoietic stem cells (HSC); LSK CD34+Flt3−CD48−CD150+, multipotent progenitors 1 (MPP1); LSK CD34+Flt3−CD48+CD150+ (MPP2); LSK CD34+Flt3−CD48+CD150− (MPP3); LSK CD34+Flt3+CD48+CD150− (MPP4); LSK CD34+Flt3−CD48−CD150− (MPP5); LSK CD34−Flt3−CD48−CD150− (MPP6) of one control mouse. (C) Representative FACS analysis and fractions of cells in TBM of CD45−CD31−Ter-119−CD63+ mesenchymal stromal cells of one control mouse. [file 40164_2025_614_MOESM1_ESM.pptx]

## Slide 1
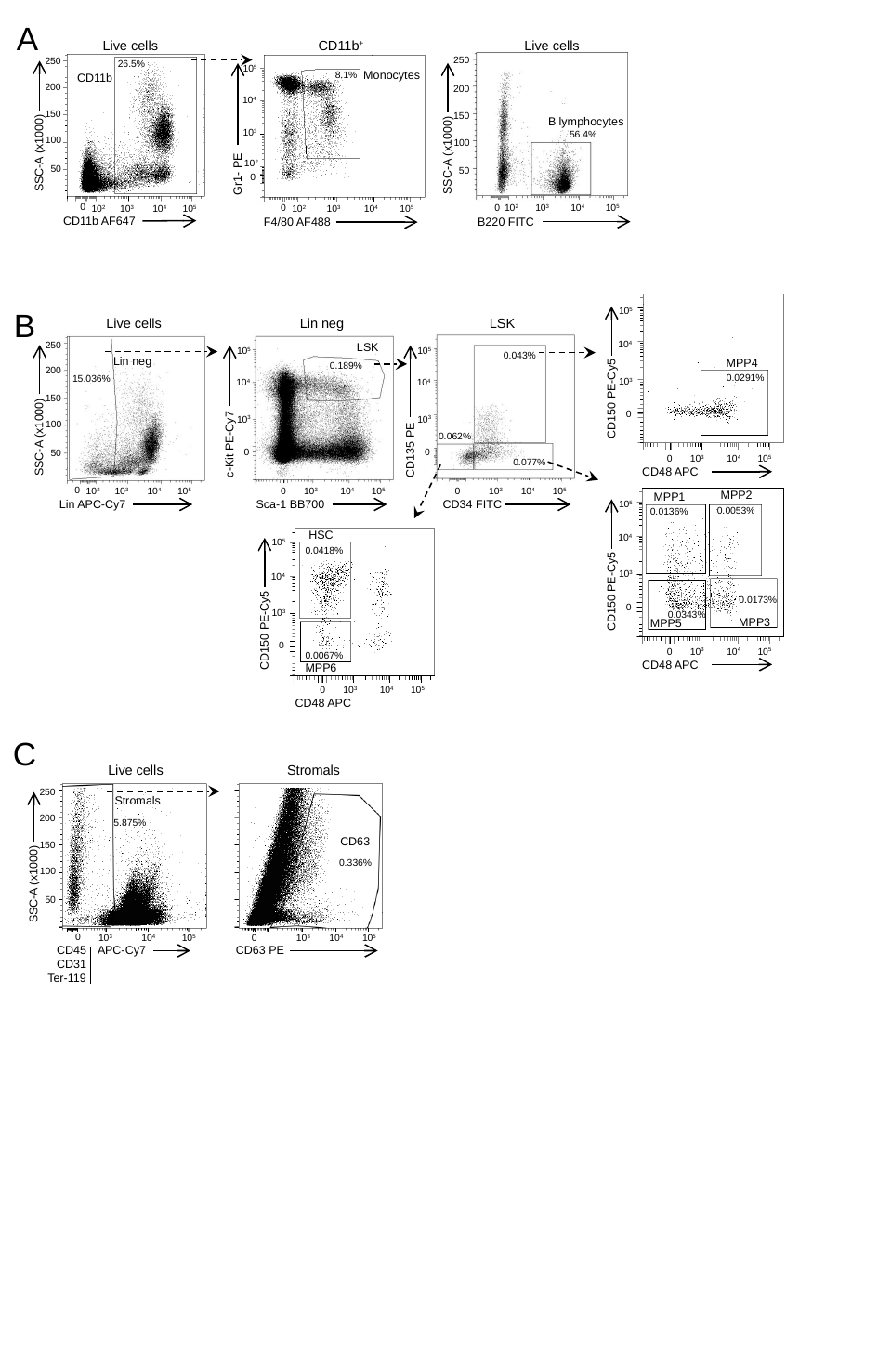

A
Live cells
CD11b+
Live cells
 250
 250
26.5%
 105
Monocytes
8.1%
CD11b
 200
 200
 104
 150
 150
B lymphocytes
SSC-A (x1000)
SSC-A (x1000)
 103
56.4%
 100
 100
Gr1- PE
 102
 50
 50
 0
 0
 0
 0
 102
 103
 104
 105
 102
 103
 104
 105
 102
 103
 104
 105
CD11b AF647
B220 FITC
F4/80 AF488
B
 105
Live cells
Lin neg
LSK
 104
 250
LSK
 105
 105
0.043%
Lin neg
MPP4
0.189%
 200
0.0291%
15.036%
 103
 104
 104
CD150 PE-Cy5
 150
SSC-A (x1000)
 0
 103
 103
 100
c-Kit PE-Cy7
CD135 PE
0.062%
 0
 0
 50
 0
 103
 104
 105
0.077%
CD48 APC
 0
 102
 103
 104
 105
 0
 103
 104
 105
 0
 103
 104
 105
MPP2
MPP1
Lin APC-Cy7
Sca-1 BB700
CD34 FITC
 105
0.0053%
0.0136%
HSC
 104
 105
0.0418%
 103
 104
CD150 PE-Cy5
0.0173%
 0
 103
0.0343%
MPP3
MPP5
CD150 PE-Cy5
 0
 0
 103
 104
 105
0.0067%
CD48 APC
MPP6
 0
 103
 104
 105
CD48 APC
C
Live cells
Stromals
 250
Stromals
 200
5.875%
CD63
 150
SSC-A (x1000)
0.336%
 100
 50
 0
 103
 104
 105
 0
 103
 104
 105
CD45
CD31
Ter-119
APC-Cy7
CD63 PE
