# Supplementary material for: Bone marrow sympathetic neuropathy is a hallmark of hematopoietic malignancies and it involves severe ultrastructural damage
Source: Exp Hematol Oncol. 2025 Mar 5;14:31. doi: 10.1186/s40164-025-00614-x (PMC11884145; doi:10.1186/s40164-025-00614-x)
Supplement: Supplementary file 2 — Additional file 2. Table S1. Individual characteristics of acute myeloid leukemia patients. [file 40164_2025_614_MOESM2_ESM.docx]

**Supplementary Table S1. Individual characteristics of acute myeloid leukemia patients.**

| Patient | Age | Sex | Cytogenic risk group | FAB Classification^a^ | Myeloblast %^b^ |
| --- | --- | --- | --- | --- | --- |
| P1 | 45 | Female | Favorable | M0-M2 | 90 |
| P2 | 66 | Male | Intermediate | M0-M2 | 30 |
| P3 | 75 | Male | Intermediate | M0-M2 | 25 |
| P4 | 59 | Female | Adverse | M4-M5 | 20 |

1. French American British classification
2. Myeloblast percentage in peripheral blood.

**Table S1: Individual characteristics of acute myeloid leukemia patients.**
Characteristics of patients used for electronic microscopy, with age (years), sex, characterization of cytogenic group using mutational profile, French American British (FAB) classification and Myeloblast percentage in peripheral blood.
